# Supplementary material for: Modeling glioblastoma heterogeneity as a dynamic network of cell states
Source: Mol Syst Biol. 2021 Sep 16;17(9):e10105. doi: 10.15252/msb.202010105 (PMC8444284; doi:10.15252/msb.202010105)
Supplement: Supplementary file 6 — Source Data for Figure 5 [file MSB-17-e10105-s004.zip › Figure5A_sourcedata/GSEA_3017/nosigdb_stateE.GseaPreranked.1622795031093/IVY_CT.html]

Details for gene set IVY\_CT[GSEA]

|  || Dataset | state63017 |
| Phenotype | NoPhenotypeAvailable |
| Upregulated in class | na\_neg |
| GeneSet | IVY\_CT |
| Enrichment Score (ES) | -0.66091824 |
| Normalized Enrichment Score (NES) | -3.4437175 |
| Nominal p-value | 0.0 |
| FDR q-value | 0.0 |
| FWER p-Value | 0.0 |
Table: GSEA Results Summary

  

Fig 1: Enrichment plot: IVY\_CT      
 Profile of the Running ES Score & Positions of GeneSet Members on the Rank Ordered List

  

| PROBE | GENE SYMBOL | GENE\_TITLE | RANK IN GENE LIST | RANK METRIC SCORE | RUNNING ES | CORE ENRICHMENT || 1 | ID3 |  |  | 6 | 0.972 | 0.0077 | No |
| 2 | LPL |  |  | 9 | 0.958 | 0.0198 | No |
| 3 | LRRN3 |  |  | 10 | 0.943 | 0.0340 | No |
| 4 | TTYH1 |  |  | 19 | 0.731 | 0.0357 | No |
| 5 | C1orf61 |  |  | 43 | 0.553 | 0.0172 | No |
| 6 | C21orf62 |  |  | 62 | 0.487 | 0.0035 | No |
| 7 | NES |  |  | 92 | 0.414 | -0.0242 | No |
| 8 | KCNIP1 |  |  | 99 | 0.402 | -0.0252 | No |
| 9 | IGFBP2 |  |  | 102 | 0.395 | -0.0215 | No |
| 10 | SEMA6A |  |  | 130 | 0.348 | -0.0479 | No |
| 11 | GATM |  |  | 149 | 0.335 | -0.0639 | No |
| 12 | DCLK2 |  |  | 169 | 0.318 | -0.0813 | No |
| 13 | FADS2 |  |  | 181 | 0.310 | -0.0895 | No |
| 14 | LRRC17 |  |  | 182 | 0.310 | -0.0848 | No |
| 15 | SDC3 |  |  | 200 | 0.295 | -0.1002 | No |
| 16 | NPAS3 |  |  | 227 | 0.280 | -0.1264 | No |
| 17 | ITGB8 |  |  | 239 | 0.268 | -0.1352 | No |
| 18 | OIP5 |  |  | 426 | -0.291 | -0.3484 | No |
| 19 | EGFR |  |  | 427 | -0.291 | -0.3440 | No |
| 20 | DNMT1 |  |  | 466 | -0.304 | -0.3838 | No |
| 21 | BCHE |  |  | 471 | -0.305 | -0.3839 | No |
| 22 | SAPCD2 |  |  | 517 | -0.319 | -0.4317 | No |
| 23 | CSPG5 |  |  | 535 | -0.328 | -0.4466 | No |
| 24 | ETV1 |  |  | 563 | -0.341 | -0.4731 | No |
| 25 | RNASEH2A |  |  | 633 | -0.378 | -0.5481 | No |
| 26 | GINS2 |  |  | 658 | -0.399 | -0.5701 | No |
| 27 | HIST1H2AM |  |  | 660 | -0.400 | -0.5652 | No |
| 28 | HIST1H2AG |  |  | 675 | -0.414 | -0.5754 | No |
| 29 | TTK |  |  | 724 | -0.454 | -0.6247 | No |
| 30 | LRRN1 |  |  | 756 | -0.490 | -0.6535 | Yes |
| 31 | CDCA3 |  |  | 759 | -0.497 | -0.6484 | Yes |
| 32 | SOX11 |  |  | 764 | -0.504 | -0.6454 | Yes |
| 33 | DBI |  |  | 776 | -0.526 | -0.6504 | Yes |
| 34 | OLIG2 |  |  | 781 | -0.538 | -0.6469 | Yes |
| 35 | BLM |  |  | 786 | -0.543 | -0.6434 | Yes |
| 36 | CDC20 |  |  | 793 | -0.562 | -0.6419 | Yes |
| 37 | DSEL |  |  | 794 | -0.563 | -0.6334 | Yes |
| 38 | ASF1B |  |  | 798 | -0.568 | -0.6284 | Yes |
| 39 | HES6 |  |  | 800 | -0.569 | -0.6209 | Yes |
| 40 | CDCA8 |  |  | 808 | -0.585 | -0.6203 | Yes |
| 41 | CDCA2 |  |  | 816 | -0.597 | -0.6195 | Yes |
| 42 | LMNB1 |  |  | 818 | -0.606 | -0.6115 | Yes |
| 43 | AURKA |  |  | 824 | -0.623 | -0.6079 | Yes |
| 44 | CKAP2L |  |  | 826 | -0.625 | -0.5997 | Yes |
| 45 | DTL |  |  | 831 | -0.637 | -0.5947 | Yes |
| 46 | BUB1 |  |  | 837 | -0.653 | -0.5907 | Yes |
| 47 | GAS1 |  |  | 839 | -0.671 | -0.5817 | Yes |
| 48 | BIRC5 |  |  | 843 | -0.679 | -0.5750 | Yes |
| 49 | CENPM |  |  | 844 | -0.682 | -0.5647 | Yes |
| 50 | DHFR |  |  | 851 | -0.701 | -0.5611 | Yes |
| 51 | KIF2C |  |  | 855 | -0.721 | -0.5537 | Yes |
| 52 | NUF2 |  |  | 856 | -0.721 | -0.5429 | Yes |
| 53 | CENPK |  |  | 857 | -0.722 | -0.5320 | Yes |
| 54 | KIF4A |  |  | 861 | -0.736 | -0.5243 | Yes |
| 55 | FBXO5 |  |  | 864 | -0.742 | -0.5155 | Yes |
| 56 | CCNB2 |  |  | 865 | -0.745 | -0.5042 | Yes |
| 57 | DLL3 |  |  | 868 | -0.762 | -0.4951 | Yes |
| 58 | NDC80 |  |  | 869 | -0.762 | -0.4836 | Yes |
| 59 | BUB1B |  |  | 871 | -0.766 | -0.4732 | Yes |
| 60 | MELK |  |  | 874 | -0.776 | -0.4638 | Yes |
| 61 | PTTG1 |  |  | 880 | -0.791 | -0.4577 | Yes |
| 62 | MCM10 |  |  | 881 | -0.798 | -0.4456 | Yes |
| 63 | KIF14 |  |  | 885 | -0.809 | -0.4369 | Yes |
| 64 | EZH2 |  |  | 886 | -0.810 | -0.4247 | Yes |
| 65 | FAM111B |  |  | 888 | -0.822 | -0.4135 | Yes |
| 66 | KIF15 |  |  | 890 | -0.834 | -0.4020 | Yes |
| 67 | OLIG1 |  |  | 891 | -0.839 | -0.3893 | Yes |
| 68 | RAD51AP1 |  |  | 894 | -0.853 | -0.3788 | Yes |
| 69 | CENPE |  |  | 899 | -0.873 | -0.3703 | Yes |
| 70 | PBK |  |  | 900 | -0.880 | -0.3570 | Yes |
| 71 | FANCI |  |  | 901 | -0.882 | -0.3437 | Yes |
| 72 | SMC4 |  |  | 902 | -0.886 | -0.3303 | Yes |
| 73 | CCNE2 |  |  | 907 | -0.895 | -0.3215 | Yes |
| 74 | GTSE1 |  |  | 912 | -0.941 | -0.3119 | Yes |
| 75 | MYBL2 |  |  | 914 | -0.959 | -0.2986 | Yes |
| 76 | CLSPN |  |  | 916 | -0.964 | -0.2852 | Yes |
| 77 | PRC1 |  |  | 919 | -0.980 | -0.2728 | Yes |
| 78 | DLGAP5 |  |  | 923 | -0.998 | -0.2612 | Yes |
| 79 | ESCO2 |  |  | 926 | -1.067 | -0.2475 | Yes |
| 80 | HIST1H2AH |  |  | 927 | -1.078 | -0.2312 | Yes |
| 81 | UBE2C |  |  | 928 | -1.108 | -0.2144 | Yes |
| 82 | NUSAP1 |  |  | 929 | -1.112 | -0.1977 | Yes |
| 83 | TPX2 |  |  | 930 | -1.124 | -0.1807 | Yes |
| 84 | NCAPG |  |  | 931 | -1.142 | -0.1634 | Yes |
| 85 | ASPM |  |  | 932 | -1.146 | -0.1461 | Yes |
| 86 | CENPF |  |  | 935 | -1.200 | -0.1304 | Yes |
| 87 | HMGB2 |  |  | 937 | -1.238 | -0.1128 | Yes |
| 88 | CDK1 |  |  | 938 | -1.248 | -0.0940 | Yes |
| 89 | RRM2 |  |  | 940 | -1.403 | -0.0740 | Yes |
| 90 | TOP2A |  |  | 942 | -1.417 | -0.0537 | Yes |
| 91 | HIST1H1D |  |  | 944 | -1.810 | -0.0276 | Yes |
| 92 | HIST1H1B |  |  | 945 | -1.903 | 0.0012 | Yes |
Table: GSEA details [plain text format]

  

Fig 2: IVY\_CT: Random ES distribution      
 Gene set null distribution of ES for **IVY\_CT**
